# Epidemiology and Clinical Outcomes of HTLV‐1: A Comprehensive Narrative Review of Endemic and Nonendemic Regions

**DOI:** 10.1155/cjid/8667755

**Published:** 2026-02-22

**Authors:** Bezhan Noori, Ramin Shahbahrami, Yousef Douzandegan, Sayed-Hamidreza Mozhgani, Mehdi Norouzi, Seyed Mohammad Jazayeri

**Affiliations:** ^1^ Department of Virology, School of Public Health, Tehran University of Medical Sciences, Tehran, Iran, tums.ac.ir; ^2^ Department of Infectious Diseases and Tuberculosis, Kabul Medical University of Sciences, Kabul, Afghanistan; ^3^ Research Center for Clinical Virology, Tehran University of Medical Sciences, Tehran, Iran, tums.ac.ir; ^4^ Department of Microbiology and Virology, School of Medicine, Alborz University of Medical Sciences, Karaj, Iran, abzums.ac.ir; ^5^ Non-Communicable Disease Research Center, Alborz University of Medical Sciences, Karaj, Iran, abzums.ac.ir

**Keywords:** ATL, endemic, HAM/TSP, HTLV-1, HTLV-1 outcomes

## Abstract

Human T‐lymphotropic virus Type 1 (HTLV‐1) is a globally distributed, oncogenic retrovirus endemic in specific regions, including southwestern Japan, sub‐Saharan Africa, the Caribbean, parts of South America (notably Brazil), Iran, and Indigenous communities in Australia. Although most infections are asymptomatic, a minority of carriers develop severe, life‐altering conditions: adult T‐cell leukemia/lymphoma (ATL) or HTLV‐1‐associated myelopathy/tropical spastic paraparesis (HAM/TSP). This narrative review presents a comprehensive analysis of epidemiological studies, clinical reports, and public health surveillance data. Data on HTLV‐1 prevalence, incidence, clinical outcomes, proviral load associations, and public health measures were extracted and compared across major endemic and nonendemic regions. HTLV‐1 exhibits extreme geographic heterogeneity. Hyperendemic foci include southwestern Japan (carrier population ∼534,000 in 2020), parts of Brazil (estimated 800,000 carriers), the Caribbean (e.g., Jamaica, general population prevalence ∼6.1%), and sub‐Saharan Africa (estimated 2–5 million infections, the largest global burden). In Central Australia, prevalence among Indigenous adults over 45 reaches 49.3%, the highest recorded regional rate globally. Prevalence varies significantly within populations: In Brazil, it is highest in the north/northeast. In Gabon, rural prevalence is 8.7%, rising to 12.5% in rainforest provinces, with Pygmy ethnicity identified as an independent risk factor. In Iran, prevalence is concentrated in the northeast (2%–7%), whereas the rest of the country shows rates below 1%. In contrast, prevalence is very low in nonendemic areas such as the United States and most of Europe (< 0.01% in Spain and Italy), except for Romania (5.3 per 10,000 donors) and areas with migrant populations. Incidence data are sparse but informative: In Japan, the annual incidence among blood donors is 6.88 per 100,000 person‐years for women and 2.29 per 100,000 person‐years for men. In the United Kingdom, the incidence of HAM/TSP among HTLV‐1 carriers is 1.98 per 1000 person‐years. A Brazilian cohort reported an HAM/TSP incidence of 1.47% over 3 years, substantially higher than Japan’s lifetime risk of 0.25%. HTLV‐1 remains a significant yet profoundly neglected global pathogen, exhibiting extreme geographic heterogeneity in prevalence, clinical outcomes, and transmission dynamics, driven by complex interactions of viral genetics, host factors, and disparities in public health infrastructure. Although proven cost‐effective interventions such as universal antenatal screening in Japan have demonstrably reduced transmission, the persistent “silent” spread in endemic, low‐resource regions particularly sub‐Saharan Africa and Indigenous Australia demonstrates a critical global health inequity demanding urgent, region‐specific strategies for screening, prevention, and patient care to mitigate its substantial burden of morbidity and mortality.

## 1. Introduction

Human​ T‐lymphotropic virus Type 1 (HTLV‐1) is a human retrovirus first isolated in 1980 [1, 2]. It carries a ∼9 kb RNA genome packaged within a complex retroviral structure and encodes both structural and regulatory proteins. The structural genes gag, pol, and env encode the matrix, capsid, nucleocapsid, reverse transcriptase, integrase, protease, and envelope glycoproteins necessary for viral assembly and entry [1, 3].

HTLV‐1 also expresses regulatory proteins (Tax and Rex) and accessory proteins (p12, p13, p30, and p21Rex), as well as the antisense gene product HBZ [4, 5]. Tax drives viral replication and cellular transformation through activation of signaling pathways including NF‐κB, CREB, and AP‐1, leading to increased proliferation and resistance to apoptosis [6], and *Rex* regulates viral mRNA expression at the post‐transcriptional level [7]. HBZ supports viral persistence, promotes infected T‐cell survival, and modulates host immunity [8].

HTLV‐1 primarily infects CD4^+^ T‐lymphocytes, although CD8^+^ T cells, dendritic cells, and monocytes can also be targets. Transmission occurs through mother to child (mainly breastfeeding), sexual contact, blood transfusion, and contaminated needles, with proviral load (PVL) and host immune status influencing transmission risk and disease progression [9].

**Figure 1:**
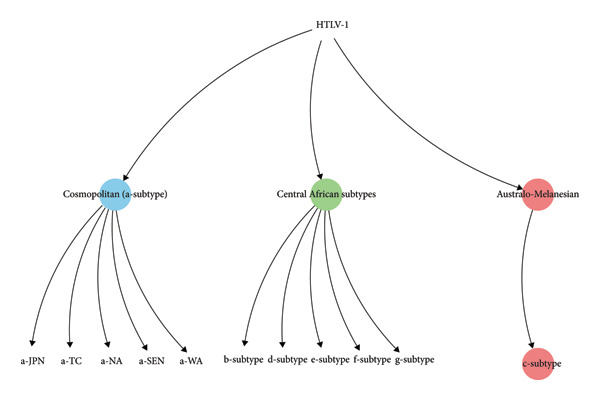
Molecular genotypes of HTLV‐1. Major genetic subtypes of human T‐lymphotropic virus Type 1 (HTLV‐1). The root represents the ancestral HTLV‐1 lineage, which diverged into three primary clades: (1) the cosmopolitan (a‐subtype) group (blue), (2) the Central African subtypes (green), and (3) the Australo‐Melanesian lineage (red). The cosmopolitan a‐subtype includes geographically defined variants such as a‐JPN (Japan), a‐TC (transcontinental), a‐NA (North America), a‐SEN (Senegal), and a‐WA (West Africa), reflecting widespread dissemination likely through human migration. The Central African clade comprises multiple distinct subtypes (b‐, d‐, e‐, f‐, and g‐subtypes), indicating high genetic diversity within this region. The Australo‐Melanesian lineage branches into the c‐subtype, predominantly found in Melanesia and parts of Australia, suggesting a long‐term, isolated evolutionary trajectory.

HTLV‐1 is classified as a neglected tropical pathogen [10], which is endemic in several hot spots worldwide, and the World Health Organization (WHO) estimated 5–10 million infected individuals globally as of 2012, although prevalence is likely underestimated due to several reasons such as limited screening and broader epidemiological studies [11, 12]. A recent systematic review and meta‐analysis estimated the global prevalence of HTLV‐1 at 0.91%. Prevalence was higher in low human development index (HDI) countries (1.18%) than in high HDI countries (0.41%). Across populations, the prevalence rates were highest in the general population (1.65%), followed by pregnant women (0.34%) and blood donors (0.04%). This pattern was consistent, with all groups showing higher prevalence in low HDI settings. Globally, HTLV‐1 prevalence was 0.91%, nearly threefold higher in low versus high HDI countries. The observed prevalence within the general population was about five times higher than in pregnant women and 41 times higher than in blood donors [13]. The distribution of HTLV‐1 molecular genotypes worldwide is a key factor in understanding viral diversity and epidemiology and is illustrated in Figure 1.

The virus high‐prevalence foci include southwestern Japan, parts of the Caribbean such as Jamaica, Trinidad, South America (notably Brazil), West and Central Africa, and some regions of the Middle East mainly in Iran [14]. Brazil alone is thought to harbor hundreds of thousands of carriers, and the African continent, with 2–5 million infections, represents the largest HTLV‐1 endemic region [15]. The virus spreads mainly through breastfeeding, sexual contact, and blood products [16, 17]. Screening blood donors or pregnant women markedly reduced transmission in several countries such as Japan [18]; however, many endemic countries have limited screening programs, allowing ongoing “silent” spreads of the virus [19].

## 2. Clinical Outcomes

Most people infected with HTLV‐1 do not develop symptoms, but about 5% develop progressive disorders [19]. The two major associated diseases of the virus are HTLV‐1‐associated myelopathy/tropical spastic paraparesis (HAM/TSP), a chronic neurological disorder, and adult T‐cell leukemia/lymphoma (ATL), an aggressive CD4+ T‐cell malignancy [20]. HAM/TSP tends to affect younger adults (mean onset 40–50 years) with spastic paraparesis, sensory changes, and bladder dysfunction [21], and ATLL usually presents later and carries a poor prognosis with a median survival of less than one year in the acute subtype [22, 23]. HTLV‐1 also causes other inflammatory conditions such as uveitis and exacerbates coinfections due to immune dysregulation [24, 25]. The risk of developing HTLV‐1‐associated disease could be different in different geographical areas [20]. The two major associated diseases of the virus are HAM/TSP, a chronic neurological disorder, and ATL, an aggressive CD4+ T‐cell malignancy [21]. HAM/TSP tends to affect younger adults (mean onset 40–50 years) with spastic paraparesis, sensory changes, and bladder dysfunction [22], and ATLL usually presents later and carries a poor prognosis with a median survival of less than one year in the acute subtype [23, 24]. In addition to these major diseases, HTLV‐1 has been implicated in a range of inflammatory and autoimmune manifestations, including uveitis, arthritis, polymyositis, Sjögren’s syndrome, and infective dermatitis, reflecting the virus’s strong immunomodulatory effects [25, 26]. The risk of developing HTLV‐1‐associated disease could be different in different geographical areas [27].

*Available data may suggest ATLL is more frequently diagnosed and reported in Japan, whereas HAM/TSP is more frequent in Brazil and the Caribbean. However, apparent differences may reflect disparities in diagnostic capacity, disease registries, and healthcare access rather than true biological variation* [28].

Most of the infected patients remain asymptomatic, and the lifetime risk of developing ATLL among HTLV‐1 carriers is estimated at approximately 2.5%–6% in certain endemic regions of Japan [24].

Notably, ATLL represents roughly 25% of peripheral T‐cell lymphomas in Asia, particularly Japan, whereas it accounts for only 2% in North America and 1% in Europe [29].

The WHO classification delineates ATLL into four distinct clinical subtypes: smoldering, chronic, lymphomatous, and acute [30]. Patients with smoldering or chronic ATLL exhibit a median overall survival of approximately 30–55 months, whereas those with lymphomatous and acute subtypes have considerably poorer prognoses, with median survival of 10 and 8 months, respectively [23].

HAM/TSP develops in approximately 3% of HTLV‐1 carriers, although the lifetime risk differs across endemic regions. In Japan, the reported lifetime risk was as low as 0.25%, whereas one cohort from Brazil suggested a substantially higher risk than the 3%, reporting an incidence of 1.47% over a median follow‐up of 3 years [31]. In the United Kingdom, a 2025 cohort study reported that the cumulative incidence of HAM among 297 HTLV‐1‐infected individuals was 1.35% (four cases), corresponding to an incidence rate of 1.98 per 1000 person‐years [32].

High HTLV‐1 PVL in blood is a major predictor of disease, and the HTLV‐1 carriers with elevated PVLs are at much greater risk for both ATL and HAM/TSP [33]. Host genetic factors and concomitant infections (HIV, HCV, and dengue) can further influence outcomes, although some studies suggest certain coinfections do not significantly alter the progression of HTLV‐1 [34]. Despite the global scope of HTLV‐1, there is not much research comparing outcomes across regions; therefore, in this study, we aim to review the findings to compare the virus prevalence, clinical manifestations, transmission patterns, risk factors, population burden, and screening efforts. The clinical profile of HTLV‐1‐associated diseases is summarized in Table 1, whereas country‐based epidemiological data, including prevalence and geographic distribution, are presented in Table 2.

**Table 1:** Clinical profile of HTLV‐1‐associated diseases: symptoms, pathophysiology, diagnosis, treatment, and prognosis.

HTLV type	Associated diseases	Main symptoms and clinical features	Pathophysiology or mechanism	Diagnostic criteria	Treatment options	Prognosis or outcomes
HTLV‐1	Malignancy: ATLL: adult T‐cell leukemia/lymphoma (acute, lymphomatous, chronic, smoldering) subtypes) [35] Neuroinflammatory: HTLV‐1‐associated myelopathy/tropical spastic paraparesis (HAM/TSP) [36] Inflammatory/autoimmune: HTLV‐1‐associated uveitis; polymyositis; chronic inflammatory arthritis; bronchiolo‐alveolitis (lymphocytic interstitial pneumonitis) [20, 37] Infective dermatitis: relapsing severe childhood eczematous dermatitis (IDH) [38, 39]	ATL: lymphadenopathy, hepatosplenomegaly, skin lesions, hypercalcemia, infections [40] HAM/TSP: progressive spastic paraparesis, sensory loss, bladder/bowel dysfunction [41] Uveitis: acute floaters and blurred vision due to granulomatous anterior uveitis (vitreous haze/opacities) [42, 43] Polymyositis: proximal weakness, ↑ elevated serum creatine kinase (CPK) [44, 45] Arthritis: chronic seronegative oligoarthritis (large joints) [46] Bronchioloalveolitis: chronic lymphocytic lung inflammation (bronchiolitis/ILD) often leading to fibrosis and bronchiectasis [47, 48] Dermatitis (IDH): severe relapsing eczematous rash (scalp/face) with secondary bacterial infection in children [49, 50]	CD4^+^ T‐cell retrovirus. Proviral integration + clonal expansion [51, 52] Tax ⟶ NF‐κB activation, ↑IL‐2/IL‐15, Bcl‐xL [53] HBZ contributes to oncogenesis [54] Chronic CTL activation drives HAM/TSP spinal cord injury [55]	HTLV‐1 infection: diagnosis by serology (ELISA screening, confirmatory Western blot) and PCR for proviral DNA [56–58] ATL: flower cells, peripheral blood cells, and bone marrow Examination is necessary for diagnosis and subclassification of ATLL [59] HAM/TSP: • Diagnosis relies on serum and CSF HTLV‐1 antibodies or PCR • A CSF‐to‐serum antibody ratio > 1 or positive CSF PCR strongly supports HAM/TSP • CSF may show elevated protein, Ig, oligoclonal bands, and lymphocytic pleocytosis • MRI often reveals spinal cord atrophy and T2 hyperintensity in lateral columns and anterior nerve roots [60–62]	ATL: combination chemotherapy (e.g., CHOP or CHOP‐like regimens) often with antiviral therapy (zidovudine + interferon‐α is recommended for leukemic forms) [63–65] Allogeneic hematopoietic stem cell transplant can be considered in younger patients [66] • Aggressive ATL (acute and lymphomatous subtypes) is treated with multiagent cytotoxic chemotherapy, which common regimens include VCAP‐AMP‐VECP or high‐intensity CHOP given every 2 weeks (CHOP‐14) [67] HAM/TSP: symptomatic management; corticosteroids may modestly slow progression. Investigational immunotherapies such as mogamulizumab [31, 68]	HTLV‐1 carriers: Most (≈95%) remain asymptomatic carriers [69]. An estimated lifetime risk of approximately 2%–5% of developing ATLL and between 0.25% and 1.8% of developing HAM/TSP [70]. ATL: very poor prognosis. Median survival ≈4–6 months (acute ATL), ≈10 months (lymphomatous); chronic/smoldering ATL has longer survival (72 months) [71] HAM/TSP: progressive; about 50% of patients become wheelchair‐dependent within 20 years of first symptoms [72]

**Table 2:** HTLV‐1 country‐based epidemiology.

Region/country	Year(s)/data	Population (type)	Sample size	Prevalence (%)	Incidence	High‐risk prevalence (%)	Transmission routes	Clinical outcomes	HTLV‐1 subtype	Risk factors	Diagnostics/screening	Notes/limitations	Ref
Brazil—multicenter cross‐sectional	2021–2023	General population (GP) (PHC attendees), people living with HIV, TB patients, pregnant women, pre‐exposure prophylaxis users (PREP)	3184	Overall prevalence 0.5% (17/3184; 95% CI: 0.3–0.8) City/subgroup heterogeneity noted Belém GP 1.1%; Vitória GP 1.7%)	—	Subgroup prevalences reported: HIV: 0.7% (6/926); TB: 0.9% (3/325); pregnant women: 0.1% (1/719); PrEP users: 0% (0/238)	Sexual transmission	—	17 confirmed HTLV seropositive (15 HTLV‐1; 2 HTLV‐2)	Multivariate predictors: age > 40 y—OR: 8.87 (95% CI: 1.82–43.10); female sex—OR: 4.60 (95% CI: 1.18–17.90); prior HCV—OR: 13.995 (95% CI: 2.37–82.51)	ELISA (MP Diagnostics HTLV‐1/II ELISA 4.0) ⟶ Western blot (HTLV blot 2.4)	Limitations: facility‐based recruitment (possible selection bias)	[73]
Brazil—Ananindeua (Pará) urban	2020–2024	Mixed community sampling + self‐referred individuals attending the municipal HTLV service (SAPEVH)	Phase 1: *n* = 228 randomly Phase 2 (2022–2024): 80 self‐referred attendance	In the community random sample: 2.7% (6/228)	—	—	—	—	—	Risk factors: Blood transfusion was the only statistically significant factor (*p* = 0.0028; OR = 4.18, 95% CI: 1.73–10.11)	Anti‐HTLV‐1/2 ELISA (Murex HTLV‐I + II, DiaSorin). confirmation via Western blot (HTLV blot 2.4 kit, MP Diagnostics) and/or RT‐qPCR	Highest prevalence of HTLV‐1 infection in an urban population in Brazil	[74]
Brazil (nationwide)	2008 (birth data)	Pregnant women, national model	> 2.9 million	Among pregnant women 0.1–1.05 (regionally)		—	—	Estimated: ATL: 120–604 cases (based on MTCT data) HAM/TSP: 8–272 (based on MTCT data)	—	—	—	Estimated 3024 new HTLV‐1 infection due to MTCT	[75]
Brazil (Pernambuco)	2018–2019	HIV‐infected adults (clinic cohort)	720	HIV + HTLV‐1/2 coinfection 1.5% HIV + HTLV‐1 coinfection: 1.3%	—	—	—	—		Age > 40, male sex, “pardo” ethnicity (majority); but no statistically significant risk factors found	ELISA + Western blot confirmation	First report of HTLV‐2 in Pernambuco; small clinic sample (HIV + only); not general population	[76]
Peru	2010–2022 (12‐year cohort)	General Indigenous population (Amazon, Shipibo‐Conibo Indigenous community)	2020	—	HTLV‐1+ associated with 3.11‐fold ↑ all‐cause mortality (95% CI: 1.58–6.10)	—	Mother‐to‐child (breastfeeding), sexual, parenteral	—	—	Female sex, older age, Indigenous ethnicity	—	Limited to one Indigenous community; not generalizable to the national level	[77]
Peru (Amazon, people with HIV)	2021–2023 (cross‐sectional pilot	HIV‐positive individuals in the Peruvian Amazon	293 PWH	HTLV positive (14/293 4.8%) including 1 HTLV‐1: 0.3%; 11 HTLV‐2: 3.8% 2 nontypeable 0.7%	—	—	Sexual, vertical, parenteral (likely overlaps with HIV transmission)	—	—	Risk factors (univariate): age ≥ 50 years strongly associated (OR: 21.9; 95% CI: 4.77–100); low education (OR: 2.96; 95% CI: [0.95–9.27] −100)	Recombinant HTLV I + II ELISA; confirmation by INNO‐LIA (line immunoassay)	Pilot study; small sample size; HIV‐focused cohort	[78]
Peru		Greater Iquitos, Peruvian (Amazon), and general population (in the review study)	300	1.7% (5/300; 95% CI: 0.7%–3.8%) pooled estimates for Peru: general population prevalence 2.9% (95% CI: 1.2%–5.3%) and prevalence in women of childbearing age 2.5% (95% CI: 1.2%–4.0%)	—	—	—	All five were asymptomatic	—	Most infected women had been sexually active before age 20	HTLV I + II ELISA recombinant v.4.0 (Wiener Lab). Confirmation: Western blot; qPCR (SYBR Green real time)	Small sample, single city (Iquitos), proviral load done retrospectively and missing for one patient, no follow‐up to implement breastfeeding avoidance or measure MTCT The paper’s systematic review/meta‐analysis (inside the same article) reports pooled estimates for Peru: general population prevalence 2.9% (95% CI: 1.2%–5.3%) and prevalence in women of childbearing age 2.5% (95% CI: 1.2%–4.0%)	[79]
Peru (Lima)	2001–2005	Pregnant women (maternity hospital)	2492	1.7% (42/2492; 95% CI: 1.2%–2.2%)	—	—	Sexual intercourse before age 20; transfusion history; male partner with STI marker	—	—	Age > 30, sexual debut < 20, partner STI markers; abortion/transfusion (borderline significant)	ELISA screening, Western blot confirmation	Limited to pregnant women in Lima (hospital‐based), not general population	[80]
Japan	2005–2006 (repeat donor cohort; follow‐up to 2011)	Repeat blood donors aged 16–69 years nationwide (seronegative at baseline)	3,375,821 HTLV‐1 seronegative donors included; 532 seroconverters identified during follow‐up	—	—	—	—	Incidence density: women 6.88/100,000 p‐yrs.; men 2.29/100,000 p‐yrs Estimated annual new infections ≈ 4190 (95% CI: 4064–4318) in adolescents/adults nationwide	—	Incidence higher in women	—	—	[81]
Japan (update)	2020–2021 (donors)	First‐time blood donors	—	Estimated carriers from measured prevalence = 534,000; birth cohort adjusted estimate = 658,000. Authors estimate ∼40% decrease in carriers vs 2006–2007	—	—	—	—	—	Age (older), sex (female); regional (Kyushu highest)	Blood donor ELISA/confirmatory; nationwide antenatal screening (pregnant women)	The prevalence in women was lower in all age strata than that predicted by each birth cohort	[82]
Gabon (rural, six provinces)	2013–2017	Adults > 15 years, rural population (Bantu and Pygmy ethnic groups)	2060	8.7% overall (179/2060; 95% CI: 7.5–10.0) Regional pockets: up to 14% in some provinces (Ogooué‐Ivindo 14%; Ogooué‐Lolo 11%	—	—	—	—		Female sex, increasing age, Pygmy ethnicity (vs. Bantu), multiple hospitalizations (> 5) were independent risk factors (multivariable analysis)	Screening by ELISA (HTLV‐1/2) ⟶ confirmatory Western blot (HTLV blot 2.4) PCR	—	[83]
Cameroon (southern rainforest hunters)	Before 2015	Hunters/persons with history of severe nonhuman primate (NHP) bites (Pygmies and Bantus)	269 individuals with NHP bite history (254 men, 15 women) matched to 269 controls from the same settlements (total ≈ 538)	8.6% prevalence among bitten persons (23/269) vs 1.5% in matched controls (4/269)	—	—	Zoonotic transmission via severe NHP bites	—	Subtype B (infections in hunters bitten by gorillas/chimpanzees) and Subtype F (bitten by small monkeys)	Severe NHP bite (severity correlated with HTLV‐1 infection risk)	Western blot serology; PCR	—	[84]
Iran (Birjand)	2017–2018	General population (border region)	3441	0.3% (95% CI: 0.12–0.48).	—	—	—	—	—	Multivariable analysis identified history of hospitalization as associated with HTLV‐1 occurrence (reported OR: 0.27, 95% CI: 0.07–0.97, *p* = 0.04)	ELISA	—	[85]
Iran (nationwide)	2024	Hemodialysis	12 studies pooled; total *N* = 1274	Pooled prevalence among hemodialysis patients: 2.37% (95% CI: 0.55–4.19)	—	Range in included studies: 0%–14% (higher values from Khorasan endemic area)	—	—	—	—	ELISA screening (anti‐HTLV‐1)/Western blot or PCR confirmation	—	[86]
Central Australia	2014–2018	Remote Aboriginal communities	Total *N* = 720 (children < 15 = 142; adults = 578)	Adults: 36.8% (213/578). Children: 3.5% (5/142). Prevalence rose with age (≥ 45 yrs: 49.3% [106/215])		—	Sexual contact inferred as principal route (age‐related rise)	Chronic lung disease, bronchiectasis, ATL, HAM/TSP	HTLV‐1 Subtype C (Australo‐Melanesian)	Increasing age (49.3% in > 45 y); high proviral load in symptomatic	ELISA (Murex) HTLV I + II, Western blot (HTLV‐I/II HTLV‐1c) PVL was assessed for all participants by real‐time polymerase chain reaction (PCR)	World’s highest known HTLV‐1 prevalence; very strong public health impact (respiratory, inflammatory diseases)	[87]
Australia (Queensland)	2018–2019 2004–2015	Hospital/registry data	Patients with HTLV‐1 testing recorded ATLL cases in Queensland (laboratory + registry review)	HTLV‐1‐positive: 2/2000 (0.1%, 95% CI: 0.02%–0.4%), ATL cases reported = 42 (national/series review; 10 (24.8%) cases from Queensland)	Crude ATL incidence (Queensland) reported: 0.025/100,000 (95% CI: 0.011–0.045) for Queensland (10/42 cases in Australia attributed to QLD in dataset)	—	—	—	—	—	Chemiluminescent microparticle immunoassay (CMIA) confirmed by Western blot	—	[88]
United States	2008–2021	Allogeneic blood donors (national)	> 75 million donations (> 18 million donors) > 13.9 M first‐time donors used for first‐time seroprevalence	Seroprevalence = 2.05 antibody positives per 100,000 donations (0.77 HTLV‐1, 1.03 HTLV‐2, 0.24 HTLV‐1/2 mixed); first‐time donors: 10.32 per 100,000	57 incident donors (25 HTLV‐1, 23 HTLV‐2, and 9 HTLV‐1/2). Incidence decreased from 0.30 (13 cases) in 2008–2009 to 0.25 (7 cases) in 2020–2021	—	—	—	—	Demographic risk factors: female donors accounted for most incident cases; differences by age, race/ethnicity, donor status, and region observed	ELISA + confirmatory (donor screening)	Very low prevalence in the general population	[89]
United States	2001–2015	ATLL cases captured via cancer registries (NPCR, SEER, NYSCR)	Total registry		State‐level age‐adjusted incidence ∼0.02–0.16 per 100,000. New York: 0.16 per 100,000 (383 cases); Hawaii: 0.16 (26 cases); Florida: 0.13 (285 cases)					Race/ethnicity (non‐Hispanic Black), place of birth (many NYC cases born in Caribbean countries), younger median age in NHB (median ∼54 y)	—	—	[90]
Europe	2003–2008	Blood donors (first‐time or repeat donors)	Survey across 23 countries	Scandinavia and Ireland: 0–0.17/10,000; France/Netherlands/United Kingdom: 0.45–0.48/10,000; Romania: 5.33/10,000 (clearly higher than other countries)	—	HTLV‐positive donors (88.6%): from endemic areas (82.3%) or declare to have a sexual partner coming from endemic areas (6.3%)	—	—	—	HTLV‐positive donors (88.6%): from endemic areas (82.3%) or declare to have a sexual partner coming from endemic areas (6.3%)	—	11/23 countries performed anti‐HTLV screening 4/11 (Scandinavian countries) performed the anti‐HTLV screening only on first‐time donors	[91]
United Kingdom	1991–2024	People living with confirmed HTLV‐1	Initially 545 HTLV‐1 asymptomatic ⟶ after exclusions 296–297 included in primary analysis	—	Cumulative incidence 1.35% (4/297); incidence rate 1.98 per 1000 person‐years (95% CI: 0.6–5.4)	—	—	HAM/TSP	—	High baseline HTLV‐1 proviral load prognostic for developing HAM (all incident cases had high PVL at first visit)	HTLV‐1 confirmed by standard serology (Western blot) and/or PVL	—	[32]
Jamaica	2019	Pregnant women	370 residual antenatal samples	1.62% (6/370)	—	—	Mother–child (breastfeeding)	—	—	—	CMIA screening and confirmatory Western blot	—	[92]
Colombia	2012–2022	Blood donors (national)	1,553,478	Overall donor seroprevalence 0.22% (3484/1,553,478)	Annual subgroup incidence rates: 2012–13 0.22%; 2014–15 0.26%; 2016–17 0.23%; 2018–19 0.25%; 2020–21 0.15%; 2022: 0.12%	—	—	—	—	—	—	—	[93]
Argentina	2005	Blood donors	123,233	(HTLV‐1/2): 0.05% (95% CI = 0.0432%–0.0704%) 0.03%–0.16% depending on the geographic location		—	—	—	—	—	ELISA, Western blot	—	[94]
Dominican Republic	2012–2017	Blood donors (Santo Domingo)	352,960	Period prevalence 0.26% (929/352,960; 95% CI: 0.24%–0.28%). (HTLV‐1/2 combined)	—	—	—	—	—	—	—	—	[95]
French Guiana	1991–2005; follow‐up 2018	HTLV‐1 diagnosed with pregnant women	307 pregnant women diagnosed with HTLV‐1; 268 observed (median follow‐up 16.7 years)	—	ATL incidence = 2.03 per 1000 HTLV‐1 carrier‐years (95% CI: 0.93–3.85); 9/268 ATLL (median 16.7 y)	—	—	—	—	ATL incidence rose in this mainly young cohort, which could indicate a regional trend or be specific to the Noir Marron population that predominated in the study	—	—	[96]

### 2.1. South America

#### 2.1.1. Brazil

Brazil harbors one of the world’s largest HTLV‐1 burdens and the highest number of HTLV‐1 infections in Latin America [97]. Estimates suggest that up to 800,000 people are infected with HTLV‐1 in Brazil [75]. Infections are unevenly distributed, with higher burdens in the north and northeast regions [73]. HTLV‐1 is more common among people with certain risk factors such as those living with HIV or tuberculosis [76, 98].

A multicenter cross‐sectional study of HTLV‐1 prevalence recently suggested that the primary mode of HTLV‐1 transmission in Brazil is possibly through the sexual route [73], which was consistent with findings from other studies conducted in Brazil [99],and it has been shown that HTLV‐1 is predominantly sexually transmitted in the city of Salvador, which was reported as the city with the highest HTLV‐1 endemicity in the country [99].

Vertical transmission of HTLV‐1 occurs mainly through prolonged breastfeeding; maternal factors such as high PVL and long nursing duration sharply increase the risk of child infection [100]. In contrast, the risk of HTLV‐1 infection from blood transfusion has been largely mitigated by mandatory donor screening and leukoreduction programs [101, 102].

A recent Central Brazil study found ∼0.8% seroprevalence in an urban adult sample [97]. Among pregnant women, seropositivity is generally low (0.32%), though it varies by region [103].

In a 10‐year survey conducted between 2007 and 2016, the average prevalence of HTLV‐1/2 infection among first‐time blood donors ranged between 0.1% and 0.2%, with city‐specific rates of 0.228% in Recife, 0.222% in Rio de Janeiro, 0.104% in Belo Horizonte, and 0.103% in São Paulo [104]. Although it should be noted that despite the value of blood donor data for surveillance, they must be interpreted with caution and complemented by population‐based or antenatal studies to estimate community prevalence more accurately.

Certain groups show much higher rates. The Brazilian HIV‐infected cohorts have reported HTLV‐1 prevalence up to ∼20% [76] and overall HTLV‐1/2 prevalence of1.24% among tuberculosis patients [98].

In Brazil, HTLV‐1 was first identified in 1986 among Japanese immigrants from Okinawa residing in Campo Grande, Mato Grosso do Sul (MS), with prevalence rates of 13% in immigrants and 8% in their descendants [105]. In a cohort of 219 Japanese immigrants and descendants in Campo Grande, MS, using ELISA and confirmatory immunoblot assay, HTLV‐1 infection prevalence was 6.8% [106], markedly higher than that observed in local blood donors (0.17%) [107], pregnant women (0.13%) [108], and African descendants in Central Brazil (0.5%) [109]. In contrast, a smaller prevalence of 2.38% was reported among Japanese immigrants in Tome‐Açu, northern Brazil [110]. A recent investigation among 1875 indigenous individuals from the Jaguapiru and Bororó villages in Dourados City, MS, identified an HTLV‐1 prevalence of 0.1% with all isolates classified as cosmopolitan subtype, transcontinental subgroup (HTLV‐1aA), and no HTLV‐2 infections detected [111].

The majority of Brazilian carriers remain asymptomatic, but a significant minority develops severe disease including ATLL and HAM/TSP. Modeling indicates that mother‐to‐child transmission (MTCT) alone could lead to 120–604 new ATL cases and up to a few hundred HAM/TSP cases per year, respectively [75].

Clinical data from the São Paulo cohort study found that HAM/TSP patients had a ∼7.3% mortality rate over follow‐up, compared to 2.9% for asymptomatic carriers. Coinfections with HIV or hepatitis C virus markedly exacerbated the prognosis and increased the HAM/TSP mortality rate [112]. Brazilian studies similar to other regions also showed that carriers who develop HAM/TSP or ATL have much higher PVL than asymptomatic carriers [113, 114]. In addition, HTLV‐1 infection predisposes to other conditions such as uveitis, dermatitis, and arthritis, although these are less often reported in the Brazilian literature. Notably, coinfection with the parasite *Strongyloides stercoralis* was reported among Brazilian patients [115]. In a Brazilian study of people living with HTLV‐1 (PLHTLV‐1), 3 of 27 experienced severe cases, requiring hospitalization for symptoms such as diarrhea, dehydration, and low albumin [116]. Some groups face higher risks than others.

Socioeconomic vulnerability indicators such as limited formal education and Black skin color have been linked to the higher prevalence of HTLV‐1/2 infection [104]. Miranda and colleagues argued that the higher HTLV‐1/2 prevalence among self‐declared Black individuals of their study [104] could possibly be attributed to the historical African origin of HTLV‐1/2 and its dissemination to Brazil through the transatlantic slave trade [101].

From a virological standpoint, a major risk factor for both transmission and disease is high maternal PVL. Paiva et al. also showed that a maternal PVL ≥ 100 copies/10^4 PBMCs and breastfeeding for ≥ 12 months were independent predictors of a child becoming infected [100].

In 2008, Brazil recorded 2,934,828 live births, with the prevalence of HTLV‐1 among pregnant women varying by region from 0.1% to 1.05%. Based on these figures, there was an estimation of 16,548 HTLV‐1‐infected women who became pregnant each year. Based on the HTLV‐1 regional prevalence and breastfeeding pattern, an estimated 3024 new HTLV‐1 infections occur annually via MTCT, of which about 2610 could be prevented through appropriate infant feeding counseling. Over time, these pediatric infections are expected to result in 120–604 cases of ATLL and 8–272 cases of HAM/TSP [75].

A recent study showed the relationship between increased PVLs associated with the death outcome was linked to the presence of HLA‐A^∗^30. HLA‐A^∗^33 and HLA‐A^∗^36 were linked to a reduced risk of disease progression in HAM/TSP patients, whereas HLA‐C^∗^ *12,* HLA*-C* ^∗^14, and HLA‐DRB1^∗^ *08 were tied to a higher risk of death. Among asymptomatic carriers, the* HLA*-C^∗^06 and* HLA*-DRB^∗^115 alleles were linked to higher PVL. In the HAM/TSP group,* HLA*-A^∗^30,* HLA*-A^∗^34,* HLA*-C^∗^06,* HLA*-C^∗^17, and* HLA*-DRB1^∗^* 09 were associated with increased PVL compared to those without these alleles [117].

Several preventive measures have been used to control the transmission of the virus among the Brazilian population. Blood donor screening (mandatory since 1993) has virtually eliminated transfusion‐related HTLV‐1 infection in Brazil [101, 102]. However, prenatal screening remains limited. Although the public health system (SUS) does not universally provide antenatal HTLV‐1 testing, official guidelines now recommend testing all expecting mothers and providing counseling if they are positive. This is important because identifying an infected mother allows interventions (notably advising against breastfeeding) that can prevent virtually all MTCT [118]. Recent research shows that routine HTLV‐1 testing for pregnant women in Brazil would be cost‐effective and could prevent many new cases [119]. Some cities now have specialized clinics that provide care and follow‐up for PLHTLV‐1, but there is a need for wider access, more education for health professionals, and stronger tracking of cases. In summary, although Brazil has taken important steps to control HTLV‐1—such as blood donor screening and some public health guidelines—researchers suggest more needs to be done. Expanding routine testing for pregnant women, improving education for healthcare workers, and reaching out to high‐risk groups would help reduce transmission and improve the lives of those affected by HTLV‐1 [118, 119].

#### 2.1.2. Other South American Countries

Peru is recognized as a major hot spot for HTLV‐1 infection, with multiple studies documenting related disease cases [80]. Reported prevalence is especially high among Japanese immigrants (around 16%) and their first‐generation descendants (about 4%) [120]. A nationwide meta‐analysis estimated the HTLV‐1 prevalence of 2.9% in the general population and 2.5% in childbearing women in Peru [79]. HTLV‐1 infection has been reported among Andean (Quechua and Aymara) and Amazonian indigenous populations in Peru [121]. Within the Shipibo‐Conibo, the third‐largest Amazonian group, prevalence has been documented at 6% for HTLV‐1 [122–124]. These indigenous people are the only indigenous population in Peru with confirmed cases of both HTLV‐1 and HTLV‐2 [123] and remain the most extensively studied group with this coinfection in the Americas [121]. More importantly for population health, longitudinal analyses from the Shipibo‐Conibo showed that HTLV‐1 is linked to substantially worse survival: Pooled data from two observational cohorts estimated a 3.11‐fold higher risk of all‐cause death at 12 years among persons with HTLV‐1 compared with those with HTLV‐2 or without HTLV infection [77].

Colombia can also be classified as an endemic region for HTLV‐I, given that population‐based estimates suggest viral prevalence surpasses the 1% threshold [125]. Early investigations demonstrated measurable circulation of HTLV‐1/2 across different regions. In Antioquia, analysis of 1.3 million donors between 2001 and 2014 reported a seroprevalence of 0.54% [126].

Screening of over 1.5 million donors at a major Colombian blood bank demonstrated a cumulative prevalence of 0.22% over the decade. The city of Bogotá and Ibagué had the highest prevalence of HTLV‐1/2 among blood donors. Regionally, Bogotá accounted for nearly half of all positive cases, followed by Ibagué, Barranquilla, and other cities, suggesting distinct urban clusters of higher prevalence [93]. In Medellín, analysis of 52,159 blood donors between 2014 and 2018 revealed an HTLV‐I/II seroprevalence of 0.176%, notably lower than prior estimates [125].

HTLV‐1 infection has been documented in multiple regions of Argentina, with the greatest concentration in the northeast, particularly in provinces adjacent to Bolivia, Paraguay, and Chile [127]. The northern provinces of Jujuy, Salta, and Tucumán show the highest prevalence, with some blood banks reporting rates of 1% or greater [128]. The nationwide analysis in 2004 demonstrated heterogeneous prevalence rates of HTLV‐1 across Argentina, with endemic areas in the north showing 0.6%–1.2% seropositivity among blood donors, compared to < 0.1% in nonendemic central regions [128]. A subsequent study in 2008 conducted in Jujuy province (northwest Argentina) provided further insights into viral diversity, identifying the HTLV‐1aA subtype in 65 isolates from individuals of Amerindian descent and the HTLV‐1aB subtype, typically associated with the Japanese subgroup, in descendants of non‐Japanese individuals living in Argentina [129]. In contrast, seroprevalence among blood donors in Central Argentina, including Buenos Aires, Córdoba, and Santa Fe, is markedly lower, ranging between 0.02% and 0.04% [130, 131].

In Jamaica, the mean HTLV‐1 seroprevalence is estimated at 6.1% (range 1.7%–17.4%) in the general population and 2%–3.8% among pregnant women and blood donors [132].

In Jamaica, HTLV‐1 infection is more common in women than men, with prevalence rising with age and multiparity [133]; during the mid‐1980s in Jamaica (1983–1985), HTLV‐1 prevalence among pregnant women was among the highest globally, reaching 3.5% [134] and 2% seroprevalence reported in 1988 for the same antenatal clinic [135].

MTCT is one of the most important transmission routes, as it is associated with the greatest risk of developing ATL [9]. A 2019 study analyzing residual antenatal samples from 370 pregnant women at the University Hospital of the West Indies found six cases confirmed as HTLV‐1‐positive corresponding to a prevalence of 1.62% [92].

Despite the routine HTLV‐1 screening for blood donors for decades in Jamaica, pregnant women are not tested. The cost is often cited as a barrier, and economic evaluations from the United Kingdom, Japan, and Brazil demonstrate that antenatal screening is cost‐effective, with Japan reducing transmission rates from 20% to 2.5% after adopting a national program [18, 136].

#### 2.1.3. Japan

HTLV‐1 remains a significant public health issue in southwestern Japan, particularly in the Kyushu–Okinawa region [137, 138]. HTLV‐1 infection, once concentrated in southern Japan, has spread to urban centers such as Tokyo and Osaka due to internal migration. In Greater Tokyo, one study reported approximately 70% of HTLV‐1 carriers to be migrants from endemic regions, with many originating from Kyushu and Okinawa. This migration might contribute to an increased prevalence of HTLV‐1 in these metropolitan areas [138].

Most Japanese people who carry HTLV‐1 acquired it from their mothers through breastfeeding or, in older generations, through blood transfusions before donor screening was introduced in 1986 [139]. In 2006–2007, there were an estimated 1.08 million carriers in Japan [82]. Recent analyses of first‐time blood donors (2020–21) and antenatal screening data indicated that, thanks to national efforts, this number dropped to about 534,000 by 2020—a decline of nearly 40% in just 14 years [82].

This decrease is largely due to government policies that require screening all donated blood and, since 2011, routine testing of pregnant women for HTLV‐1 antibodies [140]. In 2011, Japan began routine antenatal HTLV‐1 testing nationwide, which was nearly universal in 2016 [141]. The Japanese Health, Labor, and Welfare Science Research Group mainly advises that mothers with HTLV‐1 should feed their babies only with formula. However, research also suggests that breastfeeding for less than 3 months does not significantly increase the risk of infection, giving some families more flexibility [142].

Recently, a state‐transition modeling showed that antenatal screening was highly cost‐effective, preventing thousands of MTCT cases and dozens of ATL/HAM cases in Japan [143].

Among Japanese carriers, ATL is the most common serious disease linked to HTLV‐1. The lifetime risk of developing ATL is estimated at about 6%–7% for men and 2%–3% for women, and the nationwide registry (2010–11) found a median ATL onset age of ∼67.5 years. The disease comes in several forms, and recent years have seen an increase in the lymphoma type and in female cases [140, 144]. Unfortunately, ATL remained difficult to cure, and around 1000 people die from it each year in Japan [145].

The Japanese nationwide registration system “HAM‐net” in March 2012 reported that the Japanese patients diagnosed with HAM/TSP had the median age at diagnosis of 53, despite symptoms starting much earlier, at age 45. The majority of them (55.3%) were originally from the southernmost areas, specifically Kyushu and Okinawa. The most common initial symptoms included difficulty walking (81.9%), urinary problems (38.5%), and sensory disturbances in the lower limbs (13.9%) [146].

The lowest reported lifetime risk has been reported from Japan at approximately 0.25% [147], whereas in Brazil, it is substantially higher than 3%, with one cohort reporting an incidence of 1.47% over a median follow‐up of 3 years [31, 148].

In a U.K. cohort followed for up to 33 years (1991–2024), the cumulative incidence of HAM/TSP among 297 individuals with HTLV‐1 was 1.35% (four cases), corresponding to an incidence rate of 1.98 per 1000 person‐years. Notably, all individuals who developed HAM/TSP had elevated PVLs at their initial clinic visit [32].

These regional differences are likely multifactorial—influenced by host genetics, viral/phylogenetic subgroup, PVL distribution, routes/timing of transmission, and differences in surveillance, diagnostic practice, and study design—and therefore should be stated with appropriate caution.

Genome‐wide and HLA‐focused studies show that host genetics strongly influence susceptibility to HAM/TSP, with population‐specific patterns. In Japan, a large GWAS identified a specific amino acid variant in HLA‐DRB1 (DRB1‐GB‐7‐Leu) as a genetic risk factor for HAM/TSP development independent of PVL. Homozygous individuals for the leucine variant had an associated increased risk of HAM/TSP [149].

A recent study in Brazil examined an admixed cohort of 210 HAM/TSP patients and 165 asymptomatic carriers, and HLA‐A^∗^68 and HLA‐C^∗^07 were related to HAM/TSP risk, and certain alleles (C*12, C*14, and DRB1^∗^08) were linked to increased mortality, whereas HLA‐A^∗^33 and HLA‐A^∗^36 were protective against disease progression in HAM/TSP patients. Several alleles (such as A*30, C*06, and HLA‐DRB1^∗^09 alleles) were associated with higher PVL in the HAM/TSP group, which, in turn, is a known risk factor for disease [117].

The GWAS on the Japanese population study also reported that *HLA-B* ^∗^ *40:06*, *HLA-DRB1* ^∗^ *15:01*, and *HLA-DQB1* ^∗^ *06:02* were protective. Similar to the GWAS study on the Japanese population [149], it has been shown that *HLA-DQB1^∗^06:02* and *HLA-DRB1^∗^15:01* were protective in a population of African descent in Jamaica [150]. *HLA-A^∗^02* and *HLA-C^∗^08* were also reported to be protective against HAM/TSP in a Japanese population [151].

Cross‐sectional studies indicate that, in Japan, HTLV‐1 uveitis (HU) is the second most common disease associated with HTLV‐1 after ATL. Severe HU in young HTLV‐1 carriers following the onset of Graves’ disease and treatment with methimazole, despite having a low provirus load, was reported in Japanese studies [152].

Notably, HTLV‐1 carriers with comorbidities (bronchiectasis, malignancy, or prior infections) had significantly higher HTLV‐1 PVLs, indicating that such patients require close follow‐up for ATL development. In HTLV‐1‐positive rheumatoid arthritis (RA) patients, studies found higher PVLs and attenuated responses to TNF inhibitors [37, 153].

Researchers have identified several factors that can increase the risk of developing ATL or HAM/TSP, including being infected through breastfeeding, having a high viral load in the blood, older age at diagnosis, and a family history of ATL [33, 144]. A study using flow cytometry (CADM1 vs. CD7) showed that carriers with > 4 copies/100 PBMC have a markedly elevated risk of ATL [144].

Genetic subtype in Japan is almost uniformly the cosmopolitan (subgroup A) strain [154], and there is no evidence that subtype differences within Japan affect clinical outcome. Japan’s aging population means many carriers have other chronic illnesses (cardiovascular disease, diabetes, etc.), which may complicate HTLV‐1 disease, although data are limited [140, 155].

Coinfection of HTLV‐1 in infected people has also been reported in Japan. Individuals coinfected with HTLV‐1 had a higher risk of developing self‐reported liver disease (RR = 3.5, 95% CI: 1.9–6.4) and a significantly increased risk of death from liver cancer (RR = 8.2, 95% CI: 1.6–441.4) [156].

The Japanese government’s *Comprehensive Measures on HTLV-1* (established 2010) enshrined five pillars: (1) universal antenatal HTLV‐1 antibody screening (started 2010); (2) counseling services and provider training for HTLV‐1 carriers; (3) coordinated care and clinical guidelines for ATL and HAM/TSP; (4) public awareness campaigns (updated Ministry of Health website and educational materials); and (5) dedicated research funding (∼US$9M annually) [137].

Japan’s public health approach to HTLV‐1 is comprehensive. In addition to universal blood and antenatal screening, the government supports counseling, specialist care, public awareness, and ongoing research. The country’s experience shows that coordinated, long‐term strategies can successfully reduce transmission and improve the outlook for PLHTLV‐1.

#### 2.1.4. Africa

The African region, according to WHO, probably harbors the highest global burden of people infected with HTLV‐1 [157]. HTLV‐1 is highly endemic across sub‐Saharan Africa, with prevalence varying markedly by geography and population subgroup [15]. The most common African HTLV‐1 genotype is HTLV‐1b, found mainly in Central Africa [28, 158], which is the major strain in Gabon, Cameroon, the Democratic Republic of Congo, and Nigeria [159]. In Gabon and the DRC, more than 90% of HTLV‐1 strains belong to genotype b [159–161]. Other genotypes (d, e, f, and g) are rare in Central Africa, yet genotype d appears in 3%–5% of Gabonese strains [159, 162].

A meta‐analysis of 18 studies in pregnant women (*n* = 14,079) reported a pooled prevalence of 1.67%, ranging from 2.34% in western Africa and 2% in Central Africa to less than 0.5% in southern and eastern Africa [163].

Local surveys revealed high prevalence rates in rural settings. In Gabon, a nationwide rural survey using ELISA and confirmatory Western blot showed overall HTLV‐1 seroprevalence was 7.3%, with higher rates in women (9.0%) than men (5.4%), and prevalence reaching up to 12.5% in rainforest provinces [159].

An epidemiological survey conducted in rural Gabon between 2013 and 2017 reveals that HTLV‐1 remained highly endemic in rainforest regions. Among 2060 adults aged older than 15 years, plasma screening by ELISA identified 299 seropositive individuals. Confirmatory Western blot classified 136 as HTLV‐1 (6.6%). PCR amplification of the *env* and LTR regions confirmed 146 positive cases, and integrating serological and molecular results yielded a total of 179 HTLV‐1 infections, corresponding to an overall prevalence of 8.7%. Multivariable analyses identified female sex, increasing age, Pygmy ethnicity, and multiple hospitalizations (> 5) as independent risk factors, whereas prior nonhuman primate exposure was marginally associated with infection [83].

In Guinea‐Bissau, community cohorts conducted in 1990, 1997, and 2007 showed a prevalence of 4.6%–5.9%, with an incidence of 1.6–1.8 per 1000 person‐years. HTLV‐1 incidence was significantly higher among HIV‐positive individuals compared to HIV‐negative counterparts, whereas HIV incidence was not affected by HTLV‐1 infection [164].

In Ethiopia, a 2012 study found no HTLV‐1 cases among 556 outpatients at a rural hospital in the central region [165]. Another study conducted in Rwanda, a general population study, reported HTLV‐1 prevalences of 0.2% in rural areas and 0.3% in urban areas [166]. The HTLV‐1 infection data are limited for heavily populated regions of North and East Africa including the Horn of Africa [15]. Zoonotic transmission from nonhuman primates has been documented in Central African rainforest regions [83]. One study demonstrated that HTLV‐1 infection in Cameroonian hunters was associated with primate bites [84]. Healthcare‐related (nosocomial) transmission was also reported as an independent risk factor for HTLV‐1 infection. Gabonese studies found histories of multiple hospitalizations (with five hospitalizations or more) correlated with HTLV‐1 positivity [83]. Most African countries do not routinely test blood donors or pregnant women for HTLV‐1, so many people do not know they are infected [167].

The prevalence of HTLV‐1 among blood donors varied across African regions: 0%–0.1% in North Africa, 0%–2.6% in West Africa, 0.7%–6% in Central Africa, 0%–1.1% in East Africa, and 0%–0.1% in southern Africa. Among pregnant women, the highest rates were observed in Central Africa, with blood donors in East Gabon showing a prevalence of 6%, 4% in the Democratic Republic of Congo, and up to 2.6% in certain Nigerian blood donors from West Africa [15].

Both leukemia/lymphoma (ATL) and myelopathy (HAM/TSP) are seen in Africa, and there are not much published data about them. A small number of case series of HAM/TSP reports were published in the early 1990s from the Republic of South Africa (the Democratic Republic of Congo) [15]. Most locally identified ATL cases have been reported in South Africa, Senegal, Nigeria, Gabon, and, to a lesser degree, Mali [15]. In Senegal, around 20 cases were documented between 1994 and 2010 from the Principal Hospital in Dakar, Thiès Hospital’s dermatology department, and the Institut Pasteur in Dakar [15, 168]. Notably, coinfections in Africa may worsen HTLV‐1 outcomes. HTLV‐1 increases susceptibility to tuberculosis and leprosy, conditions common in some African regions [25].

Ethnicity appears to influence HTLV‐1 prevalence in Central Africa, with Pygmy populations exhibiting significantly higher infection rates, which were associated with increased independent risk of HTLV‐1 infection [83]. Comparable findings have been observed regarding HTLV‐1/2 infections among Bakola Pygmies in the ocean region of western South Cameroon, but the underlying factors contributing to this elevated prevalence in Pygmy groups remain unclear [169].

In numerous endemic areas, HTLV‐1 diagnosis is constrained by the expense and technical complexity of confirmatory tools such as Western blot, line immunoassay, and PCR. In Nigeria, for instance, the prevalence of HTLV‐1/2 among pregnant women is likely underestimated because systematic screening programs are absent [170].

According to the WHO Global Status Report on Blood Safety and Availability, among all African countries, only Seychelles reported having a national policy to test all blood donations for HTLV‐1/2 antibodies. Most other countries in sub‐Saharan Africa restrict mandatory blood screening to HIV, HBV, HCV, and syphilis, whereas HTLV‐1 is excluded due to perceived low risk, lack of standardized tests, and resource constraints [171]. The continent lacks formal HTLV‐1 guidelines and specialized centers. Recently, however, international organizations have called for increased attention, including targeted research on nosocomial and zoonotic transmission pathways [15, 172].

HTLV‐1 disproportionately affects areas with low HDI, where individuals may have limited access to healthcare services. Despite the high prevalence of HTLV‐1 in endemic regions and reaching 10%–25% among elderly women in the rural area of Gabon and the DRC, HTLV‐1‐related diseases are infrequently documented by local hospital clinicians; it seems that there is a huge under‐reporting of these diseases across the continent [15].

#### 2.1.5. Iran

HTLV‐1 is mainly found in the northeast of Iran, especially the Razavi and North Khorasan provinces with prevalence rates ranging from 2% to 7% among different groups of the study population. Even a distinct population group in Razavi Khorasan, near South Khorasan, showed a prevalence rate of 1.25% for HTLV‐1 [85]. In the rest of the country, the virus is much less common, with infection rates below 1%. In some cities such as Mashhad or Neyshabur, up to 3% of people may carry HTLV‐1 [173].

Phylogenetic analyses of HTLV‐1 across Iran have identified the Cosmopolitan Subtype A (HTLV‐1a) as the prevalent strain, with isolates clustering within the transcontinental Subgroup A. Sequencing of the Tax region from Tehran city blood donors followed by phylogenetic analysis revealed that Iranian HTLV‐1 isolates cluster within the Cosmopolitan Subtype a and transcontinental Subgroup A, showing closest genetic similarity to Japanese strains, suggesting historical introduction of HTLV‐1 via the Silk Road [174]. Although a recent study on Afghan refugees suggested that HTLV‐1 was not transmitted to the northeast of Iran via the land Silk Road, it is more likely that transmission occurred through maritime Silk Road routes or during the Mongol invasion [175].

Similarly, a cross‐sectional study of 140 HAM/TSP patients and ∼4500 asymptomatic carriers at Sena Hospital confirmed that all Iranian isolates belonged to HTLV‐1a, with positive selection pressure and unique mutations identified at positions 51, 82, 109, 172, 232, and 339 of the Tax proteins. Moreover, the sequence analysis identified mutations unique to clinical status: an AC‐specific substitution at position 22 (D ⟶ N) and a HAM/TSP‐specific substitution at position 146 (L ⟶ I) [176].

In northeastern Iran, 400 individuals from Torbat‐e Heydarieh were screened via ELISA and confirmed by immunoblot, with PCR and LTR sequencing used for phylogenetic analysis. The prevalent sequence type also clustered within the Cosmopolitan Subtype A, with a seroprevalence of 2% and the actual proviral prevalence of 1.25%, lower than neighboring Mashhad (2%–3%) and Neishabour (3.5%–5%) [177]. In another study in Golestan Province, among 4226 blood donors, eight ELISA‐reactive samples were further confirmed by Western blot, electrochemiluminescence, and nested PCR; phylogenetic analysis showed these isolates also belonged to the Cosmopolitan Subtype A, Subgroup A, sharing a common ancestor with Khorasan isolates, while overall prevalence was 0.09% [178].

Most people in Iran diagnosed with HTLV‐1‐related neurological disease are women in their midforties. The most common complaint was progressive lower‐limb weakness and spastic gait (≈72% of cases). Other frequent findings include urinary hesitancy or incontinence and lower‐limb paresthesia. Patients also showed brisk tendon reflexes, extensor plantar responses (positive Babinski), and impaired vibration/proprioception in the legs, but cranial nerves and cerebellar functions were not reported in Iranian HAM/TSP patients [179, 180]. Notably, Letafati and colleagues emphasized that pretransplant HTLV‐1 testing is “crucial” given ∼40% post‐transplant HAM/TSP risk [173].

Among Iranian ATLL patients, leukocytosis, neutropenia, and lymphocytosis were common findings. Moreover, elevated serum alkaline phosphatase and lactate dehydrogenase were reported in these patients [181].

In one study of 30 Iranian patients with ATL between 1995 and 2001 in the city of Mashhad, more than half of them had acute ATLL (53.3%) followed by lymphoma (26.7%), chronic (10%), and smoldering type (10%) [182].

Patients with ATL in Iran usually receive standard treatments such as chemotherapy, or combinations including interferon and zidovudine (AZT), which have shown good results in some cases. In a study on ATL patients from northeast of Iran, AZT/IFN‐α therapy led to a notable reduction in HTLV‐I PVL and plasma vascular endothelial growth factor (VEGF) levels, suggesting both antiviral and antiangiogenic effects [183].

In a cohort of 10 newly diagnosed chronic ATL patients, treatment with a combination of arsenic trioxide, IFN‐α, and AZT achieved an impressive response including seven complete remissions (CRs), two near‐CRs with persistent atypical lymphocytes, and one partial response [184].

Skin problems are common among Iranian ATLL patients. One study reported the cutaneous manifestations among 63% of ATLL patients admitted to Ghaem Hospital, in Mashhad city, during 1995–2004. The most frequently observed skin lesions were maculopapular eruptions (47.8%) and papular lesions (17.4%) [185].

In 2008, Yazdanpanah and colleagues identified a correlation between HTLV‐I infection and the occurrence of recurrent oral aphthous ulcers, eczema, and nongenital warts. In a subsequent study in 2009, the same researchers reported that individuals infected with HTLV‐I had a sixfold increased risk of developing skin lesions compared to the general population. This study also noted that women with HTLV‐I were more prone to skin lesions than men. Additionally, patients with HAM/TSP showed a higher likelihood of developing skin lesions, particularly during the second decade of the illness. Among various dermatological manifestations, only skin thickening showed a statistically significant association with HTLV‐I infection. Other skin conditions did not differ significantly between HTLV‐I‐positive individuals and the general population. Furthermore, the study observed a diminished adaptive skin response in HAM/TSP patients compared to the control group [186].

In another case–control study of 100 blood donors, HTLV‐1‐positive blood donors in Mashhad showed that carriers had far more skin complaints than seronegative controls (58% vs. 37%). Among the case group, aphthous stomatitis, herpes labialis, and nongenital warts were the most frequently observed diseases. In contrast, the control group most commonly exhibited herpes labialis, aphthous stomatitis, and skin tags [187].

Iranian HTLV‐1 patients have been studied for coinfections with other bloodborne or opportunistic pathogens. The data indicate that HTLV‐1/HIV coinfection is relatively uncommon in the general population. In a study of 1651 serum samples in the city of Mashhad, none of the 12 HIV‐seropositive subjects had HTLV‐1 [188]. However, another study among 20 HIV‐infected patients in Razavi Khorasan showed that three patients (15%) tested positive for HTLV‐1, who also had HBV/HCV infections [189].

Hepatitis B and C viruses also circulate at low levels in northeastern Iran. One study reported prevalence of ∼0.2% and 1.6% for HBV and HCV, respectively, and HTLV‐1/HBV coinfection in about 1.2% of them in the city of Birjand [85].

Iran’s Blood Transfusion Organization implemented routine HTLV‐1 screening in Mashhad and surrounding provinces as early as 1995 [190]. Today, seven of 31 provinces (primarily Razavi and North and South Khorasan and Gilan, West Azerbaijan, Ardabil, and Alborz) perform regular donor screening [191].

The Iranian Blood Transfusion Organization laboratories in Khorasan (Razavi, North, and South) handle most of the screening. In other regions of Iran, HTLV‐1 testing is generally performed by referral to these centers if requested. There is regional variability, and outside the seven screening provinces, HTLV testing is performed only when clinically indicated (investigation of myelopathy or lymphoma).

#### 2.1.6. Australia

In central regions such as the Northern Territory and far North Queensland, HTLV‐1—specifically the Australo‐Melanesian subtype (subtype C)—is highly prevalent among Indigenous Australians. Community‐based studies reported adult prevalence rates of 36.8%–49.3% in those over 45 years [87]. This age pattern implies predominant adult acquisition (likely sexual transmission) rather than perinatal infection [88].

The Central Australian Aboriginal adults currently have the highest recorded regional prevalence of HTLV‐1 infection worldwide [192].

In these communities, HTLV‐1 is associated with a substantial burden of disease; about 30% of infected adults developed conditions such as chronic lung disease, bronchiectasis, or symptomatic strongyloidiasis [87, 193].

Despite this impact, routine antenatal screening for HTLV‐1 has not been implemented in Central Australia, largely due to concerns about further complicating care for Aboriginal women [192].

Outside of these high‐prevalence regions, the situation is significantly different. A retrospective cross‐sectional study from Queensland, Australia, reported an estimated prevalence of 0.1% of HTLV‐1 infection and 4500 infected individuals among the population. The study also estimated 180 ATLL cases among HTLV‐1‐positive cases based on the lifetime risk of 4%–5% for developing ATL. Among 42 ATL cases reported in Australia, 10 (23.8%) were from Queensland (crude incidence rate 0.025/100,000), and across Australia, 19 deaths were attributed to ATL [88].

Despite a higher prevalence of HTLV‐1 among older Indigenous men, most of the ATL cases were observed among male and non‐Indigenous individuals, but the reasons for this difference are still unclear [88].

Notably, national blood donor surveillance from 2009 to 2018 supports the nonendemic status of HTLV‐1 in the wider population, revealing a prevalence of just 0.003%. Although national blood donor surveillance indicated an extremely low HTLV‐1 prevalence among Australian blood donors [194], the national picture may mask very high endemicity in the Indigenous communities of Central Australia [192].

In 2025, Australia released its first *Clinical Guidelines on HTLV-1 for Aboriginal Primary Health Care Settings*, providing a nationally coordinated, culturally safe, and community‐led framework. The guidelines emphasize improved access to remote, standardized testing and management pathways and targeted prevention through counseling on breastfeeding, sexual health, and transmission reduction [195].

#### 2.1.7. United States

HTLV‐1 infection is uncommon in the United States, affecting less than 0.1% of the population—substantially lower than endemic regions [90]. The prevalence of HTLV antibodies was estimated at 2.05 positive cases per 100,000 donations, comprising 0.77 cases of HTLV‐1, 1.03 cases of HTLV‐2, and 0.24 cases of coinfection with both HTLV‐1 and HTLV‐2. The prevalence was notably higher among first‐time donors, reaching 10.32 positive cases per 100,000 among more than 13.9 million screened individuals [89].

The Organ Procurement and Transplantation Network (OPTN) estimates that HTLV‐1 prevalence among U.S. blood donors ranges from 0.035% to 0.046%. Due to this low prevalence, mandatory HTLV‐1/2 screening of deceased organ donors was discontinued in the United States as of November 23, 2009 [196].

Transmission patterns in the United States mirror those seen worldwide, with infection most commonly acquired through breastfeeding, sexual contact, or parenteral exposures such as transfusions, shared needles, or transplants [197].

All U.S. blood donations have been screened for HTLV since 1988 [198], which has virtually eliminated transfusion‐related transmission. However, organ and tissue donors are not universally screened; in 2009, the OPTN discontinued mandatory HTLV testing due to the low yield. As a result, clusters of HTLV‐1 infection in the United States are almost exclusively linked to vertical or sexual transmission within high‐risk groups rather than via the blood supply [197].

ATLL is extremely rare in the United States, and almost all cases are found in individuals of African Caribbean descent or among recent immigrants. A national cancer registry analysis from 2001 to 2015 identified 2148 ATL cases, 18% of which were in New York State, with non‐Hispanic Black (NHB) individuals disproportionately affected [90].

In New York City, most ATL patients were foreign‐born (only 22.6% U.S.‐born), largely reflecting Caribbean immigration, and U.S. NHB diagnosed patients tend to be younger (median age ∼54 years) than those in Japan (mean age ∼67.5 years) [90].

In both countries, ATL shows a male predominance (slight male excess reported in U.S. cohorts) and often presents at an advanced stage, with generally poor survival outcomes [90, 140]. Although the lymphoma subtype is increasingly common in Japan, U.S. cases are mainly of the acute and lymphoma types, with indolent forms rarely observed [90, 140].

There are little data on the prevalence of HAM/TSP in the United States, mainly due to the low seroprevalence and frequent underdiagnosis [90]. In one prospective study, HAM/TSP developed in 3.7% of 160 HTLV‐1‐infected blood donors over about two years [199]. It is estimated that over 3600 people in the United States may have undiagnosed HAM/TSP [200] and most U.S. HAM/TSP cases occurred in immigrants from endemic areas or their descendants [201].

HTLV‐1‐related uveitis is likely under‐recognized in the United States due to the rarity and nonspecific symptoms. One case study in the United States of a 54‐year‐old woman with HIV experiencing 11 years of relapsing bilateral uveitis confirmed HTLV‐1 as the underlying cause of uveitis. Similar to other HAU patients, this woman presented with painless floaters and/or blurry vision, and vitreous opacities were common fundoscopic findings, along with a mild retinal vasculitis, whereas chorioretinal lesions were usually absent [201].

Malpica et al.’s Miami cohort (*n* = 195) study showed that 77% of ATLL patients were Afro‐Caribbean (median age 52). Chronic/smoldering cases were a minority (∼8%), whereas acute/lymphomatous cases comprised the majority. Hypercalcemia was also a common clinical manifestation in acute ATLL (65% of those cases). ATLL was reported to be highly aggressive in the population. Median overall survival was only 4.1 months for acute and 10.2 months for lymphomatous ATLL. In contrast, chronic/smoldering ATLL had much longer survival (median ∼72 months) [203].

First‐line treatment with azidothymidine plus interferon‐α (AZT‐IFN) achieved a 56% overall response rate (23% CR) in acute ATL, whereas intensive chemotherapy led to a 78% response (39% CR). AZT‐IFN was especially effective in chronic forms, with an 86% response in unfavorable chronic cases. Combination chemotherapy remains the mainstay for lymphomatous ATL, sometimes followed by allogeneic stem cell transplant. In one series, two patients who underwent transplant achieved progression‐free survival of 24 and 28 months, but both eventually relapsed [203].

Currently, the only mandated screening for HTLV in the United States is for blood and organ donors, with the FDA requiring all blood donations to be screened since 1988, which has nearly eliminated transfusion‐associated transmission [198]. There are no CDC guidelines for routine HTLV screening of pregnant women, sexual partners, or high‐risk groups. Although earlier CDC reports recommended counseling for known carriers, no current national agency suggests screening the general population.

#### 2.1.8. Europe

HTLV‐1 infection is generally rare across Europe, with the exception of specific hot spots. Romania stands out as the only well‐established endemic area, where routine blood donor screening has identified an HTLV‐1 prevalence of about 5.3 per 10,000 first‐time donors [91, 204, 205].

In contrast, Western Europe has a much lower background prevalence. Estimates suggest there are between 10,000 and 30,000 carriers each in the United Kingdom and metropolitan France [132, 206], although more recent analyses put the U.K. figure at approximately 36,300 [206].

Other countries such as Spain, Italy, and Germany report much lower prevalence, typically less than 0.01% of the population; for example, a study in Catalonia identified just 51 HTLV‐1/2 cases among over two million donations (≈0.0024%) [207].

Patterns of infection in Europe are heavily influenced by migration, with the majority of HTLV‐1 carriers either originating from or descending from populations in endemic regions such as the West Indies and Africa.

In the United Kingdom, most HTLV‐1 cases are among people of Caribbean or West African origin [132, 208].

Enhanced HTLV surveillance in England and Wales (2004–2013) shows that testing increased from 3581 to 7130 annually between 2008 and 2013, although most occurred in secondary care (82%), and testing in primary care remained rare. Of 9302 tests, 0.5% were reactive and 0.3% (27/9302) confirmed positive. Between 2004 and 2013, 858 people were diagnosed, predominantly female (65%), of Black Caribbean ethnicity (60%), born outside the United Kingdom (72%), and asymptomatic at diagnosis (45%). Despite increased testing, the demographic and clinical profile of diagnosed individuals has remained stable, and outside of blood donor screening, HTLV testing is uncommon except when investigating related diseases [209].

The Ireland and colleagues study also noted that HTLV is acquired through MTCT or through heterosexual contact [209].

Most recently, a 2025 study on HTLV‐1 screening of blood donations in the United Kingdom found over a 20‐year period more than 30 million blood donations in England were screened for HTLV. Under pooled testing, the annual rate of repeat‐reactive donations remained below 5 per 100,000, rising to 51 per 100,000 with individual testing and 123 per 100,000 with selective screening. They also found that the most HTLV‐infected individuals were U.K.‐born and that the infection was most likely acquired unknowingly through breastfeeding or heterosexual intercourse with individuals from endemic areas [210].

French overseas territories located in the America (Guadeloupe, Martinique, and French Guiana) are recognized endemic areas for HTLV‐1 infection [132].

A study conducted between 1989 and 1996 in Guadeloupe, a French overseas territory in the Caribbean, screened 59,426 blood donors for HTLV‐1. The study found a seroprevalence of 0.33% with a substantial decrease in overall HTLV‐I prevalence with time (from 0.47% in 1989 to 0.13% in 1996) [211].

National data on HTLV‐1 prevalence are limited outside the French overseas territories, with blood donor screening providing the primary source of information, and the overall national prevalence of HTLV‐1 among new blood donors is estimated at approximately 0.005% [212]. This makes France the country with the second‐highest HTLV‐1 prevalence in Europe after Romania, the only endemic European territory [132, 212, 213].

Approximately 50% of HTLV‐1‐positive donors in mainland France are from endemic regions, primarily the French Antilles and sub‐Saharan Africa, and about one‐third report sexual contact with partners from endemic areas as their sole identified risk factor [213].

In Spain and Italy, the infection is also concentrated among migrants from Latin America and other endemic countries, with recent reviews estimating roughly 26,000 HTLV‐1‐positive immigrants living in Italy [207, 214].

In Europe, people who inject drugs (PWID) are primarily affected by HTLV‐2 rather than HTLV‐1. For instance, a study in Estonia detected only HTLV‐2 among PWID, with a prevalence of 0.3% [215].

Sexual health clinics occasionally identify HTLV‐1, particularly among migrants; a Spanish study of STI clinics found a overal seroprevalence of 0.5%, mostly among Latin American men who have sex with men (MSM) [216].

Cases of HAM/TSP have been described in minority populations throughout Europe. An early series from Spain reported HAM/TSP in four out of 27 HTLV‐1‐infected individuals [217].

A large U.K. cohort study found a cumulative incidence of HAM/TSP of 1.35% over 33 years (1991–2024), with an incidence rate of ∼1.98 per 1000 person‐years, which is similar to Latin America and the Caribbean, and higher than in other high‐income countries [32]. In the U.K. cohort, all HAM/TSP patients were women who presented in midadulthood, with all having high baseline PVLs. Notably, no HAM/TSP cases were seen among those coinfected with HIV in this study [32]. The high prevalence of HAM/TSP among U.K. carriers likely reflects the Caribbean and West African origins of most affected individuals [149].

National registry data from 1989 to 2018 identified 369 people with HTLV‐1 in Spain, with just 12 of 369 individuals with HTLV‐1 (3.2%) coinfected with HIV. In contrast to the U.K. report, among these, HAM/TSP was diagnosed in 16% (two patients) of coinfected patients compared to 12.8% in those with HTLV‐1 alone (46/357 HIV‐neg). Coinfected individuals had more clinical complications, including pneumonia, extrapulmonary tuberculosis, and esophageal candidiasis, and were more likely to be diagnosed late, which may have contributed to more severe disease. However, the study did not find evidence that HIV coinfection increases the pathogenicity of HTLV‐1 [218].

ATLL remains uncommon in Europe and is usually seen in migrants or their descendants. The Spanish HTLV network recorded 35 ATLL cases among 451 HTLV‐1 diagnoses by 2022 [219], and in Romania, around five new cases were reported each year [220].

Reports from Spain described ATLL in relatively young patients (mean age ∼41) from diverse backgrounds, often with fewer classic features than typically seen in endemic regions. Chronic and smoldering subtypes are rare, which may reflect both limited case detection and the true rarity of these forms in Europe. Most reported cases have shown generalized lymphadenopathy, extranodal involvement, bone marrow infiltration, and opportunistic infections. The Barcelona series (2003–10) reported three ATLL cases (two acute and one lymphoma type) and also noted the absence of extreme leukocytosis, flower cells, and hypercalcemia among the patients [221].

One study reported an Italian case of a 32‐year‐old Nigerian man who presented with fever, lymphocytosis, and vesiculobullous skin lesions ultimately diagnosed as cutaneous ATLL [222].

#### 2.1.9. Caribbean

HTLV‐1 is endemic throughout the Caribbean, though prevalence rates vary significantly by country and population group. Large island‐wide surveys in the 1980s found he estimated the mean HTLV‐1 seroprevalence in the Jamaican general population is 6.1%, ranging from 1.7% to 17.4% depending on age group [133, 223]. Their study showed that HTLV‐1 seroprevalence had strong age‐ and sex‐related patterns, rising from 1.7% in males and 1.9% in females aged 10–19 years to 9.1% in men and 17.4% in women aged ≥ 70 years [133].

More recently, a focused antenatal study at the University Hospital of the West Indies (residual samples from 370 women, 2019) found six Western blot–confirmed HTLV‐1 cases (seroprevalence 1.62%), with at least two mother–child pairs testing positive on follow‐up—evidence that mother‐to‐child (breastfeeding) transmission remains active in Jamaica [92].

There are also notable regional differences within the Caribbean. For example, some rural communities in Haiti had prevalence rates around 4%, whereas urban areas tend to be lower [132].

In Martinique, a 1989 survey found 2.2% prevalence in the general population and 1.93% among antenatal patients. After the introduction of blood and antenatal screening in the 1990s, first‐time donor seroprevalence fell sharply, dropping from about 0.4% in the mid‐1990s to 0.15% by 2011–2015—a 63% reduction [224].

In the Dominican Republic, a study of 2012–17 blood donations in Santo Domingo found only 0.26% HTLV‐1/2 seropositivity [95].

Trinidad and Tobago are also considered endemic, especially among individuals of African ancestry [225]. A recent study compared 1255 MSM and 1822 heterosexual males attending a large HIV clinic in Trinidad between 2002 and 2023. Among MSM, 1.67% were coinfected with HIV‐1 and HTLV‐1, 5.1% with HIV‐1 and hepatitis B, and 0.16% with all three viruses. Rates among heterosexual males were 2.58%, 3.79%, and 0.16%, respectively [225].

Overall, adult prevalence rates in the Caribbean generally range from less than 1% to about 5%, which is higher than in nonendemic areas and similar to other global hot spots [95, 132].

The lifetime risk of developing HAM/TSP among carriers is estimated at 1%–2%, with incidence rates in Jamaica and Trinidad reaching 17–25 cases per 100,000 carriers per year [226].

In the Caribbean, ATLL overwhelmingly presents in the acute or lymphoma subtypes, with rare chronic/smoldering cases [227, 228]. The Caribbean ATLL acute and lymphomatous subtype population is characterized by complex cytogenetic abnormalities and a high incidence of central nervous system (CNS) involvement [228].

Comparisons with Japanese cohorts highlight several clinical differences. Japanese patients tend to have a higher incidence of ATLL, a male predominance, and a later peak age of onset. Differences in immune response have also been reported, with Jamaican subjects showing higher antibody titers and anti‐Tax antibodies, whereas Japanese patients exhibit higher PVLs but lower antibody titers—profiles associated with greater ATLL risk [229].

Median overall survival for acute and lymphomatous ATLL in Japan was 8.3 and 10.6 months, respectively, whereas a U.S. Caribbean cohort reported lower survival at 6.9 months and a response rate of just 32% to anthracycline‐based chemotherapy [23, 228].

Nonetheless, about 25% of Japanese patients undergoing hematopoietic stem cell transplantation achieved long‐term survival, similar to outcomes among long‐term survivors in the Caribbean cohort [228].

HTLV‐1 and tuberculosis are important clinical considerations in Haiti and other parts of the Caribbean. However, recent studies have found no evidence of a synergistic risk, with HTLV‐1 prevalence similar in TB patients and controls [230].

Carriers are also at increased risk of other infections, such as *Strongyloides stercoralis*; in Martinique, 42%–43% of ATLL patients had this parasitic infection [227], which might modify HTLV disease progression and increase susceptibility to tuberculosis [231].

Screening policies across the Caribbean have evolved. The Dominican Republic routinely screens all blood donors for HTLV‐1/2 [95], and countries such as Trinidad and Jamaica have well‐established blood safety protocols. More recently, the Pan American Health Organization (PAHO) has recommended adding HTLV‐1 testing to MTCT elimination programs, although systematic antenatal screening remains rare except in French territories [225].

Trinidad now offers HTLV‐1 testing to all HIV‐positive patients, and similar approaches are being adopted in Jamaica and other islands, but these measures often miss the majority of carriers in the general population. Although blood donor screening is now widespread and has improved transfusion safety, there remains a need for broader antenatal screening to further reduce transmission risk, as emphasized in recent PAHO guidelines [225].

## 3. Discussion

The marked geographic heterogeneity of HTLV‐1 infection reflects a complex interplay of viral, host, and environmental factors. Differences in viral genotypes are known to influence transmission efficiency and disease outcome; for instance, the cosmopolitan subtype (a) predominates in Japan, South America, and parts of Africa, whereas the Central African subtype (b) and Melanesian subtype (c) circulate in other endemic regions. These genetic variations may partly account for disparities in clinical progression, such as the lower lifetime risk of HAM/TSP observed in Japan compared with Brazil or the Caribbean.

Host genetic background also plays an important role. Polymorphisms in immune‐response genes—particularly HLA Class I and II alleles—have been linked to differences in PVL and disease susceptibility. For example, protective alleles such as HLA‐A02 and HLA‐Cw08 are more prevalent in Japanese carriers, possibly contributing to the lower frequency of ATL and HAM/TSP compared with populations lacking these alleles. Additionally, coinfections (e.g., *Strongyloides stercoralis* or *Mycobacterium tuberculosis*) and nutritional or environmental stressors may further amplify immune activation and viral replication, accelerating disease onset in resource‐limited settings.

Socioeconomic and healthcare disparities also significantly shape HTLV‐1 outcomes. In Japan, nationwide antenatal screening and public‐awareness programs have effectively reduced MTCT, whereas in sub‐Saharan Africa and Indigenous Australian communities, limited diagnostic capacity, lack of awareness, and insufficient blood donor screening continue to drive silent transmission. Under‐recognition and misdiagnosis of HTLV‐1‐associated conditions, particularly in nonendemic regions, contribute to the underestimation of disease burden and delayed patient care.

In summary, the observed regional differences in HTLV‐1 prevalence and clinical outcomes likely result from the convergence of viral subtype distribution, host genetic variability, environmental influences, and inequities in healthcare infrastructure. Future research should prioritize genomic and immunological comparisons across populations, coupled with the implementation of region‐specific public health interventions to reduce transmission and improve clinical management of HTLV‐1‐associated diseases.

## 4. Limitation

A major limitation lies in population sampling and selection bias. Much of the empirical evidence is derived from blood donor screening, antenatal surveillance, and clinic‐based cohorts rather than probability‐based community surveys. Blood donor data systematically exclude groups at higher risk (including older adults, marginalized populations, and people with comorbidities), whereas antenatal cohorts under‐represent older age groups who carry the greatest burden of HTLV‐1‐associated diseases. These sampling frames constrain generalizability and can yield biased prevalence estimates when extrapolated to whole populations.

Diagnostic and laboratory heterogeneity also complicates interpretation. Studies employed differing pipelines, and confirmatory testing was unevenly available across regions. This variability increases the risk of both false positives (in the absence of confirmatory testing) and false negatives (where assays had inadequate sensitivity), while hindering comparability between settings. Standardized, quality‐assured diagnostic algorithms are essential for robust surveillance.

Inconsistent outcome ascertainment and under‐reporting of HTLV‐1‐related diseases further limit the evidence base. Incidence and survival data for ATLL and HAM/TSP are often drawn from single‐center case series, specialist registries, or modeling exercises rather than population‐based incidence studies. Underdiagnosis and limited registry infrastructure—particularly in African and other low‐resource settings—likely lead to underestimates of disease burden and hinder reliable regional comparisons.

Temporal and policy heterogeneity introduces additional complexity. Programmatic interventions, such as staggered implementation of universal blood donor screening and antenatal testing, alongside demographic changes related to migration and urbanization, have shifted transmission dynamics over time. Consequently, prevalence and incidence estimate from different periods reflect distinct public health contexts, and cross‐period comparisons require careful adjustment for these secular and programmatic shifts.

## 5. Future Direction

To address these limitations, large, probability‐based, age‐stratified prevalence surveys are needed in understudied regions such as Africa, the Caribbean, Latin America (beyond Brazil), the Middle East, and Indigenous Australian communities, employing standardized diagnostic protocols to generate accurate prevalence and age‐specific burden estimates. Expansion of longitudinal cohorts with harmonized protocols across diverse settings is essential to measure incidence and the dynamic interplay of PVL, host genomics, and coinfections.

Strengthening diagnostic capacity and adopting standardized testing algorithms—combining sensitive screening ELISA, confirmatory INNO‐LIA/Western blot, and targeted PCR/PVL quantification—will reduce misclassification and improve comparability across regions. Parallel efforts should integrate social determinant research and implementation science to quantify how breastfeeding practices, sexual behaviors, stigma, and health system barriers sustain transmission and to rigorously evaluate context‐adapted interventions. Successful examples such as Japan’s antenatal screening program and Brazil’s blood donor screening provide programmatic models for adaptation elsewhere.

Broader genomic and coinfection research is also a priority, including multiethnic GWAS, viral sequencing across endemic regions, and systematic studies of interactions with HIV, TB, Strongyloides, and HBV/HCV. Finally, investment in regional ATL and HAM/TSP registries, with standardized case definitions and laboratory linkages, will improve incidence estimates, support clinical research, and enable evaluation of therapeutic strategies in the populations most affected.

## 6. Conclusion

HTLV‐1 remains a profoundly neglected global pathogen whose burden is concentrated in discrete, often marginalized populations—from Indigenous Australians and southwestern Japan to the Caribbean, parts of South America, and sub‐Saharan Africa—where millions remain undiagnosed due to absent or fragmented screening. The persistence of this epidemic is not biological inevitability but a consequence of global health inequity and policy neglect; controlling HTLV‐1 demands urgent, region‐specific integration of screening, counseling, and care into national health systems, coupled with investment in longitudinal research to quantify its impact and evaluate interventions.

## Consent

The authors have nothing to report.

## Disclosure

The authors unanimously approved the final version of the manuscript.

## Conflicts of Interest

The authors declare no conflicts of interest.

## Author Contributions

It is hereby acknowledged that all authors have accepted responsibility for the manuscript’s content and consented to its submission. They have meticulously reviewed all results.

Bezhan Noori and Ramin Shahbahrami contributed equally to this work.

## Funding

No funding was obtained for this manuscript.

## Data Availability

Data sharing is not applicable.
